# Fine mapping *qGL2H*, a major locus controlling grain length in barley (*Hordeum vulgare* L.)

**DOI:** 10.1007/s00122-020-03579-z

**Published:** 2020-03-19

**Authors:** Calum Watt, Gaofeng Zhou, Lee-Anne McFawn, Chengdao Li

**Affiliations:** 1 Western Barley Genetic Alliance, Murdoch University, Murdoch, WA Australia; 2 Western Australian State Agricultural Biotechnology Centre, Murdoch University, Murdoch, WA Australia; 3 Department of Primary Industry and Regional Development, South Perth, WA Australia

## Abstract

**Key message:**

A major grain length QTL on chromosome 2H was fine mapped to a 140.9 Kb region containing three genes.

**Abstract:**

Increasing yield is an important target for barley breeding programs. One approach to increase yield is by enhancing individual grain weights through the regulation of grain size. Fine mapping major grain size-related quantitative trait loci is necessary for future marker-assisted selection strategies, yet studies of this nature are limited in barley. In the present study, we utilised a doubled haploid population derived from two Australian malt barley varieties, Vlamingh and Buloke, coupled with extensive genotypic and phenotypic data from three independent environments. A major grain length locus identified on chromosome 2H designated *qGL2H* was fine mapped to a 140.9 Kb interval. *qGL2H* was able to account for 25.4% of the phenotypic variation for grain length and 10.2% for grain yield. Underlying *qGL2H* were three high-confidence predicted genes. One of these genes encodes a MYB transcription factor and represents a promising candidate for further genetic research.

**Electronic supplementary material:**

The online version of this article (10.1007/s00122-020-03579-z) contains supplementary material, which is available to authorized users.

## Introduction

“Yield is king,” a sentiment held by many cereal crop breeders across the globe with yield representing the most important driver of grower profitability. Changing climate, rising global population and reduction in arable land area necessitate the need to increase cereal yield potentials beyond what are currently achieved in order to meet future demand, which is expected to more than double by 2050 (Molotoks et al. 2018). Barley is the fourth most important cereal grain crop grown globally in terms of both production and area cultivated (FAOSTAT 2019). It is an important stockfeed and the primary grain used for beer and spirit production (Wendt et al. 2016). Changes to climatic conditions threaten barley production with yield modelling indicating average yield losses of up to 17% as a result of the increased frequency and severity of heat stress events alone. In comparison, growth chamber experiments have indicated this reduction could be as high as 52% when coupled with the increased average global temperature (Ingvordsen et al. 2018; Xie et al. 2018). Like all small grained cereal crops, barley yields are a reflection of two key phenotypic components: number of grain per m^2^ and individual grain weight (Fan et al. 2006; Xing and Zhang 2010). Yield improvements through pre-breeding and breeding have largely been driven by the optimisation of flowering time and increases in harvest index, enabling lodging resistance and more efficient grain filling (Hill and Li 2016; Prince et al. 2001; Sharma et al. 2018). Despite this, year-on-year yield improvements have begun to stagnate because desirable allelic combinations have largely been captured in current cultivars, and this yield growth stagnation is reflected in increasing numbers of undernourished people globally (FAOSTAT 2019; Ray et al. 2012; Schauberger et al. 2018; Xu et al. 2018a). In some regions, yield growth rates are already decreasing due to recent changes in climatic conditions resulting in poorer cultivar adaptation. Alternative traits to target are therefore necessary in order to improve the yield potential of barley.

Grain size is a major determinant of individual grain weight and has higher heritability than yield per se making it a desirable alternative trait to target. Grain size is not only important from a yield perspective but is also important from a processing standpoint. For example, maltsters prefer a plump grain because it has a more desirable starch/protein ratio, improving the efficiency of alcohol production (Agu et al. 2007). Increasing grain size is therefore an efficient and necessary strategy by which to increase yield and improve grain quality, and has been a recent focus of pre-breeding research (Walker et al. 2013; Watt et al. 2019; Wendt et al. 2016; Xu et al. 2018a).

Grain size characteristics are complex polygenic traits that are also strongly influenced by environment (Sun et al. 2013; Walker and Panozzo 2016; Xu et al. 2018a). Optimising the most desirable allelic combinations into elite backgrounds can be rapidly achieved through the use of tightly linked molecular markers and marker-assisted selection (MAS) coupled with speed breeding approaches (Watson et al. 2018). Identification and mapping QTL for grain size-related characteristics have been achieved in various genetic backgrounds (Walker et al. 2013; Watt et al. 2019; Xu et al. 2018a; Zhou et al. 2016). In many instances, the identified QTL share close homology to grain size-related genes identified in wheat and rice resulting in promising putative candidates for further research, although research of homologous gene regions in barley is restricted to a small number of publications (Bélanger et al. 2014; Wang et al. 2019; Watt et al. 2019; Xu et al. 2018a). Despite our current understanding of QTL regions contributing to grain size, only one study has fine mapped a major QTL and identified a potential candidate gene (Watt et al. 2019). While analysis of homologous sequence regions between barley and the other widely studied cereal crop species provides a promising starting point for grain size research, independent linkage and association mapping studies are still necessary to validate gene regions and identify desirable alleles underlying these homologs that will improve grain size in barley (Walker and Panozzo 2016; Zhou et al. 2016).

Quantitative trait loci mapping studies have successfully identified significant QTL on all seven barley chromosomes reported to control grain size-related characteristics (Marquez-Cedillo et al. 2000; Mather et al. 1997; Matthies et al. 2012; Walker et al. 2013; Watt et al. 2019; Xu et al. 2018a; Zhou et al. 2016). Although the limiting step is not QTL identification, the process of fine mapping and identifying the underlying candidate gene are responsible for trait variation. This is followed by validation of gene effect and characterisation of desirable alleles in different genetic backgrounds. Development of tightly linked molecular markers to underlying candidate genes and identification of desirable alleles is necessary for effective MAS. Diagnostic molecular markers have been identified for numerous traits such as waterlogging tolerance (Zhang et al. 2016); malting quality (Gong et al. 2013; Xu et al. 2018b) and disease resistance (Dinglasan et al. 2019; Zantinge et al. 2019). Despite numerous studies having mapped significant QTL associated with grain size characteristics on all seven barley chromosomes, limited research fine mapping these regions has impacted the development of diagnostic molecular markers for breeding purposes. A previous study by the authors represents the only research to date with a specific focus on fine mapping a major grain size-related locus, designated *qGL5H* (Watt et al. 2019). Using the same genetic population as Watt et al. (2019), this study describes the fine mapping result of a major grain length QTL identified on chromosome 2H that was able to explain a high proportion of the phenotypic variation for grain length and yield. An underlying gene identified in the fine-mapped interval represents a promising target for future genetic research, and the development of diagnostic flanking molecular markers in this study has potential use in MAS.

## Materials and methods

### Plant material

The population consisted of 306 DH lines derived from a cross between Vlamingh and Buloke, two-rowed spring barley varieties with distinctly different grain shapes. This is the same genetic population used by Watt et al. (2019).

### Field trials

Phenotypic data from three independent and rainfed field trials (Esperance 2016; South Stirling 2016; Wongan Hills 2017) were used for this study and represented contrasting environments in the Western Australian wheatbelt. Briefly, each genotype was sown in a 1 × 5 m^2^ plot, reduced to 3 m in late August of each season. Genotypes were partially replicated with a minimum of one replication and maximum of four in each field trial. Commercial varieties acted as controls. There were two applications of fertiliser, the first at seeding and the second when the crop had begun to tiller at Zadoks growth stage 21 (Zadoks et al. 1974). Biotic stress management varied depending on the severity of infestation and control followed standard agricultural practice for the western region. Growing season rainfall and temperature records were maintained for inclusion as covariates during statistical analysis.

### Measurement and analysis of grain size characteristics

Grain size characteristics were measured using an SC6000R digital image analyser (Next Instruments, Condell Park, Australia) using the same protocol as Watt et al. (2019). Yield (t/ha) was determined post-harvest. Grain length, width and thickness were measured on a 20–25 g sub-sample of grain.

Best linear unbiased predictions (BLUPs) for grain size characteristics and yield were calculated using linear mixed models for individual trials and a combined analysis of all three field trials known as a multi-environment trial (MET), the purpose of which was to remove environmental effects. The simplified model is given by

$${\varvec{y}}={\varvec{X}}{\varvec{\tau}}+{\varvec{Z}}{\varvec{u}}+{\varvec{e}}$$

where ***y*** is the vector of observations for different grain size characteristics; ***X ***is a design matrix associated with a vector of fixed effects ${\varvec{\tau}}$; Z is a design matrix associated with a vector of random effects ***u***; and ***e*** is the vector of residuals that include residual error variance associated with autoregressive spatial correlations in the row and column directions (Smith et al. 2019). Linear mixed models using advanced restricted maximum likelihood techniques to obtain trait BLUPs were ran using the R software package ‘ASReml’. Statistically significant differences between individuals and trials for the phenotypic measurements collected were determined using Students *t* tests using the R software, which was also used for principle component analysis (PCA).

Genotyping and QTL analysis.

Genomic DNA was isolated from grain samples using an adapted method from Ahmed et al. (2009) where instead of a phenol/chloroform/isoamyl alcohol protein degradation step, samples were placed in a 65 °C water bath for 1 h to denature any protein. Following denaturing DNA was precipitated using ethanol, spun at 4000 RPM to pelletise DNA prior to resuspension. Insertion–deletions (InDels) between Vlamingh and Buloke were determined within the QTL region identified from initial whole-genome QTL mapping activities using the BarleyVar database (in-house database) and used for fine mapping on a total of 306 DH lines. Primers were developed using the barley cv. Morex reference genome sequence in the Geneious v10.2.3 software (Kearse et al. 2012; Mascher et al. 2017).

PCR reactions were performed using freshly extracted genomic DNA from leaves in a total volume of 10 µL containing 1 µL of 10 × buffer and GC buffer, 0.25 mM dNTPs, 0.2 µM of each primer, 50 mM MgCl_2_, 50 ng genomic DNA and 0.2 U *Taq*-polymerase. The PCR protocol for gel markers was as follows: 95 °C for 3 min, 38 cycles of 94 °C for 20 s, 55–57 °C (primer-dependent) for 20 s, 72 °C for 20 s and a final extension at 72 °C for 5 min. Standard InDel markers were separated in 2% agarose gels in 0.5x TBE buffer and visualised under UV light.

Initial whole-genome QTL mapping analyses used a total of 619 DArT, SSR and SNP markers after removal of unmapped markers as outlined in a previous publication by Watt et al. (2019). The genetic map for this initial analysis was developed using JoinMap5 as described by Watt et al. (2019). Composite interval mapping (CIM) algorithms were used to conduct initial whole-genome scans using individual field trial BLUP data in MapQTL v6, culminating in a separate analysis for each field trial. LOD scores were calculated based on 1,000 permutations at a cut-off *P*-value of 1.0e-08, a minimum walk speed of 1 cM and a threshold LOD value for linkage significance of 3.5. Identified QTL were further investigated using the more sensitive multi-QTL mapping (MQM) algorithm by adding more QTL sequentially until no new loci were detected with similar threshold criteria to CIM. Percentage of phenotypic variation explained by each QTL was estimated as the R^2^ (coefficient of determination). The linkage map and identified QTL were drawn using the MapChart software (Voorrips 2002). Detected QTLs that were stable across two or more environments and had similar genetic positions (overlapping) were considered as singular loci. Any QTL that explained > 10% of the phenotypic variation was considered a major locus.

QTL fine mapping and candidate gene annotation.

Initial whole-genome QTL analysis using individual trial BLUP data identified three overlapping intervals for *qGL2H*. Fine mapping of *qGL2H* was then achieved by saturating the interval delineated by the two outermost flanking markers identified with InDel markers. In total, 95 InDel markers were designed and combined into the genetic map with further QTL analyses using MET-BLUP phenotypic data and then carried out using the same approach described previously. We used the MET-BLUP data during fine mapping to ensure that we captured the stable effect of *qGL2H*. Following marker development and subsequent QTL analysis with the newly developed InDel marker set were able to narrow down the interval. In the newly identified *qGL2H* interval, we then identified DH lines that were recombining and used Student’s *t* tests to compare the phenotype of recombinants that fell into two distinct groups to validate allelic effects at this locus. Candidate genes in the fine-mapped interval were identified using the BarleyVar database by inputting the physical positions of the QTL flanking markers. Relative gene expression profiles were investigated using The Barley Genome Explorer (BARLEX) database which is the combination of the barley cv. Morex reference sequence and high- and low-confidence gene predictions (Beier et al. 2017; Colmsee et al. 2015; Mascher et al. 2017).

## Results

### Phenotypic summary

**Fig. 1:**
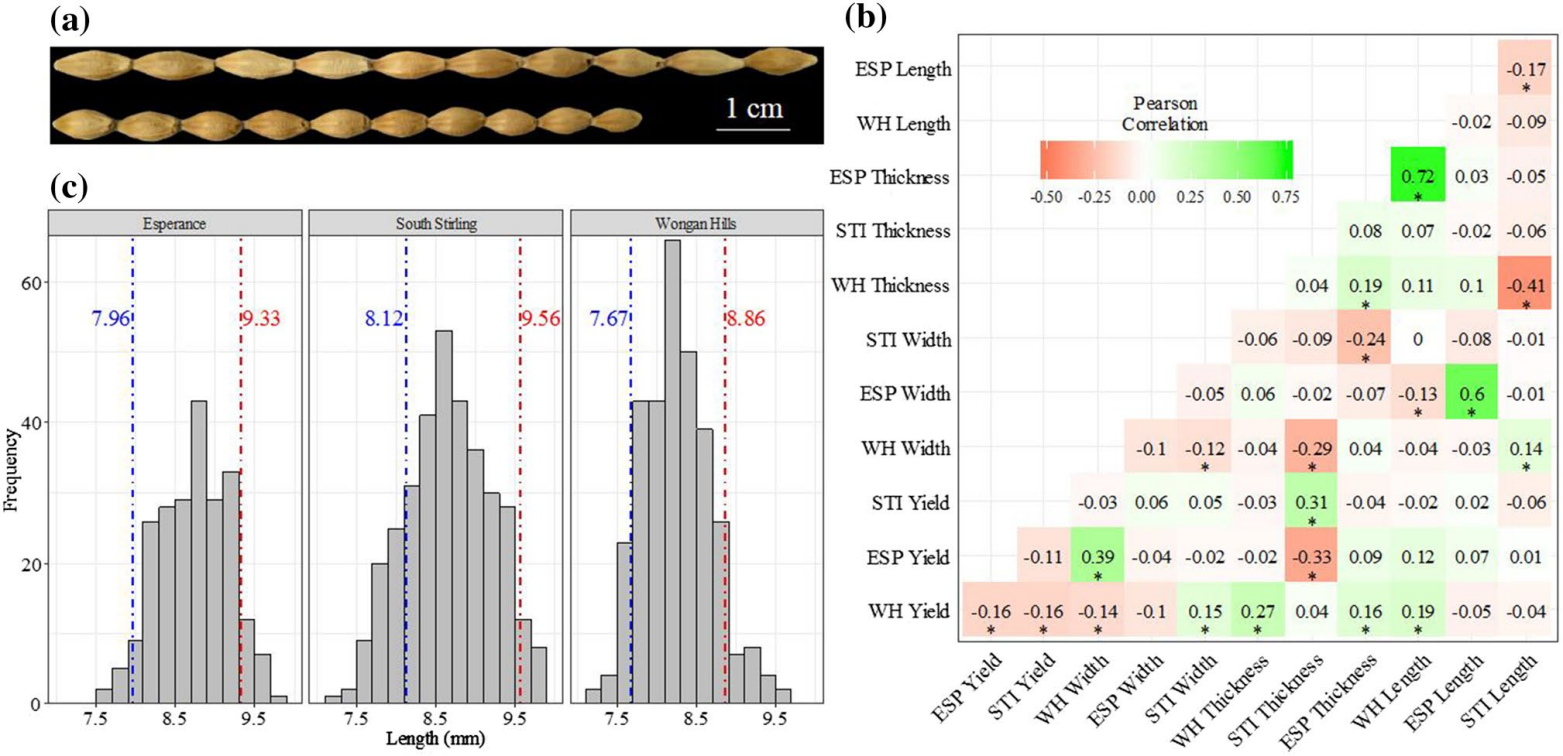
**a** Grain length of the two parents Buloke (top) and Vlamingh (bottom). **b** Pearson’s correlation coefficients between yield and three grain size characteristics measured across each field trial, * indicates significant correlation at *p* = 0.05. ESP: Esperance 2016; STI: South Stirling 2016 and WH: Wongan Hills 2017. **c** Distribution of grain length in each field trial. Vertical lines indicate average grain length of parents: Vlamingh (blue) and Buloke (red)

Grain size characteristics were normally distributed in each individual field trial after removal of outliers as expected for polygenic and quantitatively inherited traits. Phenotypic differences between the two parental lines, Vlamingh and Buloke, are shown in Fig. 1a. Transgressive segregation was evident for grain length in all trials (Fig. 1c). Correlation between grain size traits and yield varied significantly, driven by strong environmental effects (Fig. 1b). At the individual trial level, grain length was significantly positively correlated with yield in the Wongan Hills field trial, but there were no significant correlations between grain length and yield in either Esperance or South Stirling trials. Broad sense heritability ranged from 0.27 at Wongan Hills to 0.81 at both Esperance and South Stirling, respectively. The low heritability in Wongan Hills was believed to be driven by heterogeneous field variation that was not properly captured by the model and is reflected in low accuracy of BLUPs for this site despite the high replication of control varieties.

Principal component analysis using combined trial MET-BLUP data indicated that length was the most discriminating grain size trait measured within this population (Fig. S1). The PCA clearly indicates two distinct groups along the first principle component which is most correlated with grain length, where groups are based on the parental allele present at *qGL2H* and indicate that lines with the Buloke allele tend to be longer on average. Predicted values from combined trial MET-BLUP analysis indicated that all traits were significantly correlated apart from width and yield, which is inconsistent with the correlations detected in the raw field trial data (Fig. 1b). There were significant negative correlations between length and the other three traits compared. Grain thickness was significantly positively correlated with width and yield. Grain length was significantly positively correlated with yield in the Wongan Hills trial. Wongan Hills representing a harsher environment than South Stirling and Esperance, with grain length likely contributing more to yield than grain width and thickness, were more important contributors to yield in the more favourable growing environments of Esperance and South Stirling.

### Identification and mapping of QTL

Using the two-stage linkage mapping procedure in MapQTL (CIM followed by MQM), 15 significant QTLs were detected for the grain size traits only, and none for yield using whole-genome marker data and individual trial BLUPs. Of these QTL, nine were associated with grain length, a conclusion support by PCA where width and thickness are not large drivers of grain size diversity within this population (Fig. S1). Significant QTL were detected on all chromosomes aside from 3 and 7H. Chromosomes 2H and 5H were hotspots with a total of 11 QTL detected. Major loci that were detected in two or more environments (consensus) were only identified on chromosomes 2H and 5H using the individual trial BLUP data (Table 1). In this study, we were only able to detect two consensus major QTL regions, both of which regulated grain length, one being *qGL5H* and the other *qGL2H* (Table 1). These loci were identified in each individual trial QTL analysis reflecting the heritability and stability of this trait in this population.

**Table 1:** Consensus genomic regions harbouring significant QTL for grain length using whole-genome marker data

Chr	Env	Pos. (cM)	LOD	R2	Effect	Higher	Marker	Physical position	Name
Grain length (mm)									
2H	3	124.6–179.4	4.44–9.44	8.5–16.7	0.12–0.22	Buloke	2651–1774–6117–1507	47.3–577.6	*qGL2H*
5H	3	48.5–80.0	5.22–8.48	10.1–14.9	0.13–0.21	Buloke	2146–2256–3928–513	393.2–518.1	*qGL5H*

Chr, chromosome ID; Env, Number of environments detected; Pos, genetic position; LOD, logarithm of the odds; R^2^, % trait variation explained by the QTL; Effect, mean locus effect; Higher, parent contributing discriminating allele; Marker, QTL discriminating flanking markers; Physical position, based on barley reference genome in Mb; and Name, of locus

^*^Consensus; an overlapping chromosomal region detected in two or more field trials and considered to be the same locus

### Fine mapping of 2H major grain length QTL region

**Fig. 2:**
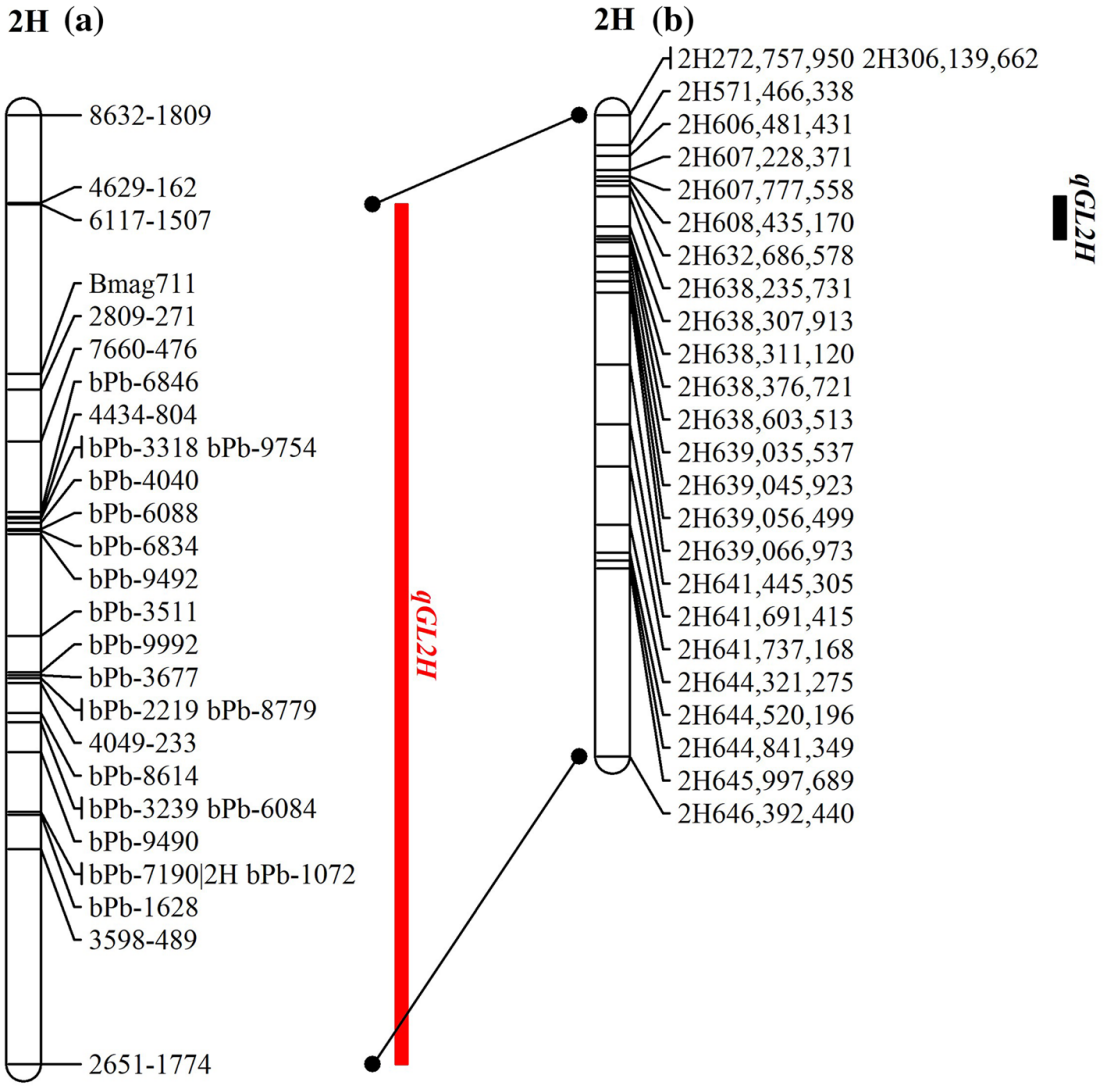
Fine mapping result of *qGL2H*. **a** Subset of whole-genome marker data and consensus QTL interval (red) detected during initial whole-genome mapping and **b** genetic map created using InDel markers (Table S1) and associated fine-mapped QTL region (black)

**Fig. 3:**
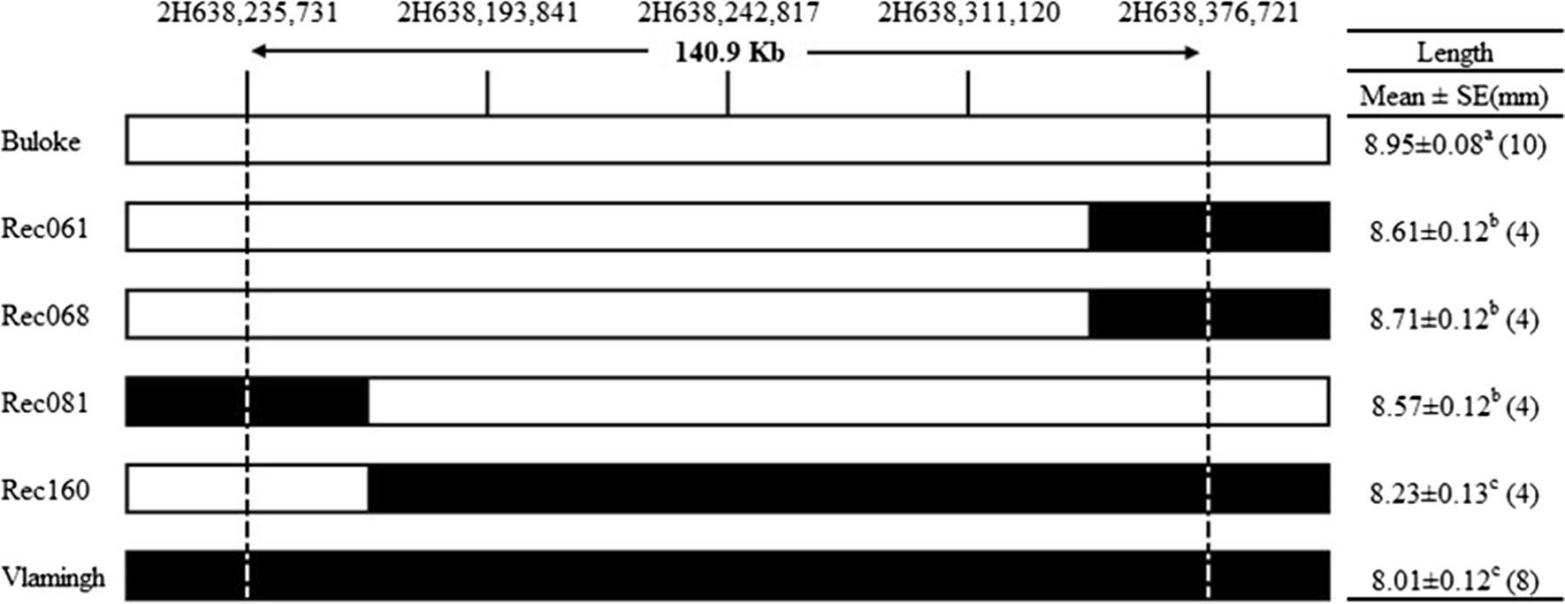
Genotypes and phenotypes of parents and recombinant DH lines using BLUP data for grain length. Genetic structure depicted as white (homozygous Buloke) and black (homozygous Vlamingh) in *qGL2H* (dash) using a subset of the 95 interval markers. All recombinant lines had Buloke alleles between *qGL5H* flanking markers. Table on the right indicates variation in grain length; numbers in brackets indicate number of plots of each genotype and letters depict significant differences at *p* = 0.05

Two significant major grain length QTL were identified using combined trial MET-BLUP data in this population, one each on chromosome 2H and 5H. The interval identified on chromosome 2H overlapped with the major consensus region *qGL2H* identified through individual trial QTL analyses (Table 1). Using MET-BLUP data, the LOD scores for *qGL2H* and *qGL5H* were 22.7 and 20.9, respectively. The percentage of phenotypic variation was 25.4% and 21.6% for *qGL2H* and *qGL5H,* respectively. Furthermore, a previous study found a strong QTL for grain length in a similar location to *qGL2H* making it a suitable candidate for fine mapping (Wang et al. 2019). Whole-genome QTL analyses using individual trial BLUP data indicated that *qGL2H* was flanked by SNP markers 2651–1774 and 6117–1507, which based on the barley cv. Morex reference sequence is a large interval spanning 530.3 Mb (Table 1). Analysis using MET-BLUP data found *qGL2H* accounted for 25.4% of phenotypic variation for grain length with a LOD score of 22.7. No other grain size characteristic was influenced by *qGL2H* which was consistent with initial whole-genome mapping results. Interestingly, *qGL2H* was also able to explain 10.2% of the phenotypic variation for grain yield when using the MET-BLUP data, a relationship not observed during individual trial QTL analyses. To fine map *qGL2H*, we designed 95 polymorphic InDel markers saturating this 530.3 Mb interval and used the combined trial MET-BLUP data to undertake successive QTL analyses and marker development to continuously narrow down the target region. Using this dataset which consisted of the entire DH population being genotyped, we were able to fine map *qGL2H* to a 140.9 Kb interval between markers 2H638,235,731 and 2H638,376,721 representing a substantial reduction in the size of this interval (Figs. 2 and 3, S2). At a population level, DH lines that have the Buloke allele at both of these flanking markers have an average grain length of 8.78 mm compared to 8.47 mm for those with Vlamingh alleles which are significantly different at *p* = 0.001. Of the population, four recombinant DH lines in total were identified within the *qGL2H* interval (Fig. 3), three of which had Buloke alleles between the flanking markers (Rec061, Rec068 and Rec081) and in each individual trial had grains that were significantly longer than the recombinant line with Vlamingh alleles, Rec160. This is supported by an effect at this locus indicating that the Vlamingh allele reduces grain length upwards of 0.22 mm in this population (Table 1).

Within this newly mapped interval, three high-confidence predicted genes were identified, *HORVU2Hr1G089310*, a predicted MYB transcription factor of subgroup 15, which is a promising candidate as it has been reported to be involved in cell cycle control and cell division in the longitudinal direction (Qi et al. 2018; Tombuloglu et al. 2013; Wu et al. 2017). The other predicted genes were *HORVU2Hr1G089320* (hexosyltransferase) and *HORVU2Hr1G089330* (root UV-B sensitive 2 protein). Relative gene expression indicates that of the three candidate genes, the MYB transcription factor has the highest expression of the three genes in tissues associated with the developing inflorescence (Fig. S3).

## Discussion

Linkage mapping is an efficient method to identify the genetic control of polygenic traits such as grain length. In the present study, Vlamingh and Buloke, two elite Australian two-rowed malting varieties with contrasting grain lengths were used to generate a DH population to fine map a major grain length locus that overlapped with one previously identified on chromosome 2H (Watt et al. 2019). Grain length was normally distributed in each environment as expected for a polygenic and quantitatively inherited trait (Lai et al. 2017; Sadeghzadeh et al. 2010; Vafadar Shamasbi et al. 2017). Correlation between the three grain size characteristics varied from weakly positive to negative in each trial, indicating that there is a significant genotype by environment interaction occurring. Within individual field trials, grain length was only significantly correlated with grain width in the Esperance environment (Fig. 1b). For the most part, grain plumpness characteristics, width and thickness tended to be significantly positively correlated with yield in individual environments apart from grain width at Wongan Hills which was negatively correlated with yield (*p* = 0.05). A possible reason for this distinction is Esperance and South Stirling are milder environments compared to Wongan Hills, enabling a genotypes maximum grain width and thickness to fully express, whereas in the harsher Wongan Hills environment grain length would become a larger contributing factor for grain yield as supported by the significant positive correlation between the two (Fig. 1b).

Watt et al. (2019) previously identified 23 significant QTLs for grain yield and three grain size characteristics of which two represented major loci controlling grain length in this population. One designated *qGL5H* was previously fine mapped to a 1.7 Mb interval that was able to explain 21.6% of the phenotypic variation for grain length. In this present study, we fine mapped the second major grain length QTL originally identified, designated *qGL2H* to a 140.9 Kb interval containing three high-confidence predicted genes. Consistent with studies in wheat and rice, grain length QTL tend not to coincide with other grain size-related characteristics such as width and thickness. This is due to the fact that grain length is to a large extent controlled at the cellular level by cell elongation and proliferation in the longitudinal direction in developing endosperm and husk tissues (Rabiei et al. 2004; Yu et al. 2017). In contrast, grain width and thickness correlate primarily with endosperm cell proliferation in the transverse direction (Segami et al. 2016; Wang et al. 2015). In the present study, *qGL2H* did not coincide with other grain size-related characteristics; however, it did represent a major locus for grain yield explaining 10.2% of the phenotypic variation for this trait using MET-BLUP data but not individual trial BLUPs. This result is likely to be driven by the reduced shrinkage of BLUPs towards the mean when running a MET compared to individual trial analyses. Interestingly, a previous study identified significant QTL for grain length, a similar region to *qGL2H,* although they also led to variation in other grain size-related characteristics such as grain width (Wang et al. 2019). The fine mapping result of *qGL2H* indicates there are two QTL regions near one another on chromosome 2H contributing to grain length variation; however, it is evident that due to the genetic background of each population, neither were able to be identified simultaneously.

Three high-confidence predicted genes are located within *qGL2H*. A promising candidate for the control of grain length is *HORVU2Hr1G089310* which encodes a MYB transcription factor protein of subgroup 15. Previous research in other cereal species has indicated that transcription factors are heavily involved in the control of grain size through regulation of cell proliferation and differentiation (Arora et al. 2017; Gong et al. 2018; Ji et al. 2019; Qi et al. 2018). Specifically, a MYB transcription factor designated *GL4* was shown to regulate cell elongation in the outer and inner glumes of African rice which directly regulated grain length (Wu et al. 2017). In the present study, comparisons between the three putative candidate genes indicated that the MYB transcription factor has the highest relative expression in developing inflorescence tissues of the lemma and palea compared to the other two genes (Fig. S3). Wu et al. (2017) found that in rice, a premature stop codon in the coding region of this MYB transcription factor was responsible for the significant differences in grain length observed. Interestingly, the MYB transcription factor gene located within *qGL2H* shows approximately 90.37% DNA sequence homology to *SH4*, a MYB-like protein encoding gene associated with non-shattering in Asian rice that is orthologous to *GL4*. A review of the current literature did not find any clear link between the hexosyltransferase and root UV-B sensitive protein encoding genes and any grain size-related characteristic, further reinforcing that the MYB transcription factor is the likely candidate gene underlying *qGL2H*.

Watt et al. (2019) previously conducted whole-genome QTL analysis to identify loci controlling yield and three components contributing to grain size (length, width and thickness). The present study fine mapped *qGL2H*, which was found to significantly associate with grain length and yield. We identified one promising candidate gene that based on current research is likely to regulate grain size, annotated as *HORVU2Hr1G089310* and encoding a MYB transcription factor. MYB-like proteins represent one of the largest families of transcription factors and have been linked to numerous biological functions, including abiotic stress tolerance and control of grain size characteristics through regulation of cell division and differentiation within certain developmental tissues (Qi et al. 2018; Wu et al. 2017; Xiong et al. 2014). While the other two candidate genes cannot be ruled out, the MYB transcription factor represents the most promising avenue on which to focus further research. In lieu of further research into these genes, the two flanking InDel markers of *qGL2H* could be highly useful for MAS as they are diagnostic for grain length and yield within this population. As it stands, *qGL2H* represents a promising genetic region with which yield and grain length can be manipulated simultaneously.

## Electronic supplementary material

Below is the link to the electronic supplementary material.

Supplementary file1 (DOCX 179 kb)

Supplementary file2 (DOCX 59 kb)

Supplementary file3 (DOCX 41 kb)

Supplementary file4 (XLSX 110 kb)

Supplementary file5 (XLSX 20 kb)

## Abbreviations

MET: Multi-environment trial
BLUP: Best linear unbiased prediction
DArT: Diversity array technology
MQM: Multi-QTL mapping
QTL: Quantitative trait loci
CIM: Composite interval mapping
PCA: Principal component analysis
DH: Doubled haploid
InDel: Insertion–deletion
SSR: Simple sequence repeat
SNP: Single nucleotide polymorphism

## References

[CR1] Agu RC, Brosnan JM, Bringhurst TA, Palmer GH, Jack FR (2007). Influence of corn size distribution on the diastatic power of malted barley and its impact on other malt quality parameters. J Agric Food Chem.

[CR2] Ahmed I, Islam M, Arshad W, Mannan A, Ahmad W, Mirza B (2009). High-quality plant DNA extraction for PCR: an easy approach. J Appl Genet.

[CR3] Arora S, Singh N, Kaur S, Bains NS, Uauy C, Poland J, Chhuneja P (2017). Genome-Wide Association Study of grain architecture in wild wheat *Aegilops tauschii*. Front Plant Sci.

[CR4] Beier S, Himmelbach A, Colmsee C (2017). Construction of a map-based reference genome sequence for barley, *Hordeum vulgare* L. Sci Data.

[CR5] Bélanger S, Gauthier M, Jean M, Sato K, Belzile F (2014). Genomic characterization of the *Hordeum vulgare* DEP1 (*HvDEP1*) gene and its diversity in a collection of barley accessions. Euphytica.

[CR6] Colmsee C, Beier S, Himmelbach A, Schmutzer T, Stein N, Scholz U, Mascher M (2015). BARLEX – The Barley draft genome explorer. Mol Plant.

[CR7] Dinglasan E, Hickey L, Ziems L, Fowler R, Anisimova A, Baranova O, Lashina N, Afanasenko O (2019). Genetic characterization of resistance to *Pyrenophora teres* f. *teres* in the international barley differential Canadian lake shore. Front Plant Sci.

[CR8] Fan C, Xing Y, Mao H, Tingting Lu, Han B, Caiguo Xu, Li X, Zhang Q (2006). *GS3*, a major QTL for grain length and weight and minor QTL for grain width and thickness in rice, encodes a putative transmembrane protein. Theor Appl Genet.

[CR9] FAOSTAT (2019) Worldwide cereal production comparisons. Food and Agriculture Organization of the United Nations Statistical Database, https://www.fao.org

[CR10] Gong R, Cao H, Zhang J, Xie K, Wang D, Yu S (2018). Divergent functions of the GAGA- binding transcription factor family in rice. Plant J.

[CR11] Gong X, Westcott S, Zhang X-Q, Yan G, Lance R, Zhang G, Sun D, Li C (2013). Discovery of novel *Bmy1* alleles increasing β-amylase activity in chinese landraces and tibetan wild barley for improvement of malting quality via MAS. PLoS ONE.

[CR12] Hill CB, Li C (2016). Genetic architecture of flowering phenology in cereals and opportunities for crop improvement. Front Plant Sci.

[CR13] Ingvordsen CH, Lyngkjær MF, Peltonen-Sainio P, Mikkelsen TN, Stockmarr A, Jørgensen RB (2018). How a 10-day heatwave impacts barley grain yield when superimposed onto future levels of temperature and CO_2_ as single and combined factors. Agric Ecosyst Environ.

[CR14] Ji X, Du Y, Li F, Sun H, Zhang J, Li J, Peng T, Xin Z, Zhao Q (2019). The basic helix-loop- helix transcription factor, *OsPIL15*, regulates grain size via directly targeting a purine permease gene *OsPUP7* in rice. Plant Biotechnol J.

[CR15] Kearse M, Moir R, Wilson A (2012). Geneious basic: an integrated and extendable desktop software platform for the organization and analysis of sequence data. Bioinformatics.

[CR16] Lai Y, Yu Y, Liu X, Wan H, Zhang Z, Wang L, Leng Y, Ma L, Yang W, Feng Z (2017). Association mapping of grain weight, length and width in barley (*Hordeum vulgare*) breeding germplasm. Int J Agric Biol.

[CR17] Marquez-Cedillo LA, Hayes PM, Jones BL, Kleinhofs A, Legge WG, Rossnagel BG, Sato K, Ullrich SE, Wesenberg DM, Project NABGM (2000). QTL analysis of malting quality in barley based on the doubled-haploid progeny of two elite North American varieties representing different germplasm groups. Theor Appl Genet.

[CR18] Mascher M, Gundlach H, Himmelbach A (2017). A chromosome conformation capture ordered sequence of the barley genome. Nature.

[CR19] Mather DE, Tinker NA, LaBerge DE, Edney M, Jones BL, Rossnagel BG, Legge WG, Briggs KG, Irvine RB, Falk DE, Kasha KJ (1997). Regions of the genome that affect grain and malt quality in a North American two-row barley cross. Crop Sci.

[CR20] Matthies IE, van Hintum T, Weise S, Röder MS (2012). Population structure revealed by different marker types (SSR or DArT) has an impact on the results of genome-wide association mapping in European barley cultivars. Mol Breed.

[CR21] Molotoks A, Stehfest E, Doelman J, Albanito F, Fitton N, Dawson TP, Smith P (2018). Global projections of future cropland expansion to 2050 and direct impacts on biodiversity and carbon storage. Global Change Biol.

[CR22] Prince SD, Haskett J, Steininger M, Strand H, Wright R (2001). Net primary production of U.S. midwest croplands from agricultural harvest yield data. Ecol Appl.

[CR23] Qi L, Ding Y, Zheng X, Xu R, Zhang L, Wang Y, Wang X, Zhang L, Cheng Y, Qiao W, Yang Q (2018). Fine mapping and identification of a novel locus *qGL12.2* control grain length in wild rice (*Oryza rufipogon* Griff.). Theor Appl Genet.

[CR24] Rabiei B, Valizadeh M, Ghareyazie B, Moghaddam M, Ali AJ (2004). Identification of QTLs for rice grain size and shape of Iranian cultivars using SSR markers. Euphytica.

[CR25] Ray DK, Ramankutty N, Mueller ND, West PC, Foley JA (2012). Recent patterns of crop yield growth and stagnation. Nat Comm.

[CR26] Sadeghzadeh B, Rengel Z, Li C, Ha Y (2010). Molecular marker linked to a chromosome region regulating seed Zn accumulation in barley. Mol Breed.

[CR27] Schauberger B, Ben-Ari T, Makowski D, Kato T, Kato H, Ciais P (2018). Yield trends, variability and stagnation analysis of major crops in France over more than a century. Sci Rep.

[CR28] Segami S, Yamamoto T, Oki K, Noda T, Kanamori H, Sasaki H, Mori S, Ashikari M, Kitano H, Katayose Y, Iwasaki Y, Miura K (2016). Detection of novel QTLs regulating grain size in extra-large grain rice (*Oryza sativa* L.) lines. Rice.

[CR29] Sharma R, Draicchio F, Bull H, Herzig P, Maurer A, Pillen K, Thomas WTB, Flavell AJ (2018). Genome-wide association of yield traits in a nested association mapping population of barley reveals new gene diversity for future breeding. J Exp Bot.

[CR30] Smith AB, Borg LM, Gogel BJ, Cullis BR (2019). Estimation of factor analytic mixed models for the analysis of multi-treatment multi-environment trial data. J Agric Biol Environ Stat.

[CR31] Sun L, Ma D, Yu H, Zhou F, Li Y, Luo L, Gao G, Zhang Q, Xu C, He Y (2013). Identification of quantitative trait loci for grain size and the contributions of major grain-size QTLs to grain weight in rice. Mol Breed.

[CR32] Tombuloglu H, Kekec G, Sakcali MS, Unver T (2013). Transcriptome-wide identification of R2R3-MYB transcription factors in barley with their boron responsive expression analysis. Mol Genet Genomics.

[CR33] Vafadar Shamasbi F, Jamali SH, Sadeghzadeh B, Abdollahi Mandoulakani B (2017). Genetic mapping of quantitative trait loci for yield-affecting traits in a barley doubled haploid population derived from clipper × sahara 3771. Front Plant Sci.

[CR34] Voorrips RE (2002). MapChart: Software for the graphical presentation of linkage maps and QTLs. J Hered.

[CR35] Walker CK, Ford R, Munoz-Amatriam M, Panozzo JF (2013). The detection of QTLs in barley associated with endosperm hardness, grain density, grain size and malting quality using rapid phenotyping tools. Theor Appl Genet.

[CR36] Walker CK, Panozzo JF (2016). Genetic characterisation, expression and association of quality traits and grain texture in barley (*Hordeum vulgare* L.). Euphytica.

[CR37] Wang Q, Sun G, Ren X, Du B, Cheng Y, Wang Y, Li C, Sun D (2019). Dissecting the genetic basis of grain size and weight in barley (Hordeum vulgare L.) by QTL and comparative genetic analyses. Front Plant Sci.

[CR38] Wang S, Li S, Liu Q (2015). The *OsSPL16-GW7* regulatory module determines grain shape and simultaneously improves rice yield and grain quality. Nat Genet.

[CR39] Watson A, Ghosh S, Williams MJ (2018). Speed breeding is a powerful tool to accelerate crop research and breeding. Nat Plants.

[CR40] Watt C, Zhou G, McFawn L-A, Chalmers KJ, Li C (2019). Fine mapping of *qGL5H*, a major grain length locus in barley (*Hordeum vulgare* L.). Theor Appl Genet.

[CR41] Wendt T, Holme I, Dockter C, Preuß A, Thomas W, Druka A, Waugh R, Hansson M, Braumann I (2016). *HvDep1* is a positive regulator of culm elongation and grain size in barley and impacts yield in an environment-dependent manner. PLoS ONE.

[CR42] Wu W, Liu X, Wang M (2017). A single-nucleotide polymorphism causes smaller grain size and loss of seed shattering during African rice domestication. Nat Plants.

[CR43] Xie W, Xiong W, Pan J, Ali T, Cui Q, Guan D, Meng J, Mueller ND, Lin E, Davis SJ (2018). Decreases in global beer supply due to extreme drought and heat. Nat Plants.

[CR44] Xing Y, Zhang Q (2010). Genetic and molecular bases of rice yield. Annu Rev Plant Biol.

[CR45] Xiong H, Li J, Liu P, Duan J, Zhao Y, Guo X, Li Y, Zhang H, Ali J, Li Z (2014). Overexpression of *OsMYB48-1*, a novel MYB-related transcription factor, enhances drought and salinity tolerance in rice. PLoS ONE.

[CR46] Xu X, Sharma R, Tondelli A, Russell J, Comadran J, Schnaithmann F, Pillen K, Kilian B, Cattivelli L, Thomas WTB, Flavell AJ (2018). Genome-wide association analysis of grain yield-associated traits in a Pan-European barley cultivar collection. Plant Genome.

[CR47] Xu Y, Zhang X-Q, Harasymow S, Westcott S, Zhang W, Li C (2018). Molecular marker-assisted backcrossing breeding: an example to transfer a thermostable β-amylase gene from wild barley. Mol Breed.

[CR48] Yu J, Xiong H, Zhu X (2017). *OsLG3* contributing to rice grain length and yield was mined by Ho-LAMap. BMC Biol.

[CR49] Zadoks JC, Chang TT, Konzak CF (1974). A decimal code for the growth stages of cereals. Weed Res.

[CR50] Zantinge J, Xue S, Holtz M, Xi K, Juskiw P (2019). The identification of multiple SNP markers for scald resistance in spring barley through restriction-site associated sequencing. Euphytica.

[CR51] Zhang X, Zhou G, Shabala S, Koutoulis A, Shabala L, Johnson P, Li C, Zhou M (2016). Identification of aerenchyma formation-related QTL in barley that can be effective in breeding for waterlogging tolerance. Theor Appl Genet.

[CR52] Zhou H, Liu S, Liu Y, Liu Y, You J, Deng M, Ma J, Chen G, Wei Y, Liu C, Zheng Y (2016). Mapping and validation of major quantitative trait loci for kernel length in wild barley (*Hordeum vulgare* ssp*. spontaneum*). BMC Genet.

