# Supplementary material for: Fine mapping qGL2H, a major locus controlling grain length in barley (Hordeum vulgare L.)
Source: Theor Appl Genet. 2020 Mar 19;133(7):2095–103. doi: 10.1007/s00122-020-03579-z (PMC7311499; doi:10.1007/s00122-020-03579-z)
Supplement: Supplementary file 1 — Supplementary file1 (DOCX 179 kb) [file 122_2020_3579_MOESM1_ESM.docx]

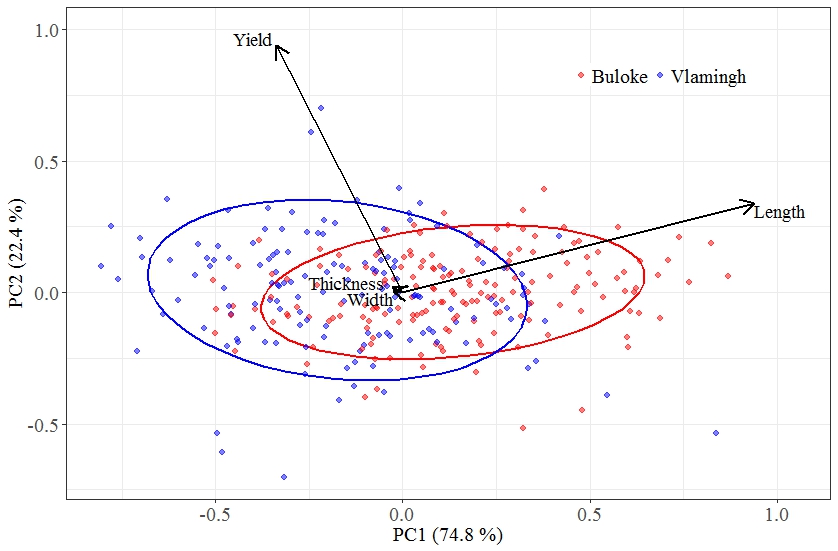


**Fig. S1** Unscaled two-dimensional PCA using BLUPs from MET analysis, groupings based on parental allele at major QTL region. First two principle components account for 97.2 % of variance between individuals
