# Supplementary material for: Fine mapping qGL2H, a major locus controlling grain length in barley (Hordeum vulgare L.)
Source: Theor Appl Genet. 2020 Mar 19;133(7):2095–103. doi: 10.1007/s00122-020-03579-z (PMC7311499; doi:10.1007/s00122-020-03579-z)
Supplement: Supplementary file 2 — Supplementary file2 (DOCX 59 kb) [file 122_2020_3579_MOESM2_ESM.docx]

**Fig. S2** Fine mapping result of *qGL2H* using the InDel markers designed in the interval between flanking markers 2651-1774–6117-1507 for grain length. X-axis represents Mb and horizontal dashed line a LOD threshold of significance of 3.0
