# Supplementary material for: Fine mapping qGL2H, a major locus controlling grain length in barley (Hordeum vulgare L.)
Source: Theor Appl Genet. 2020 Mar 19;133(7):2095–103. doi: 10.1007/s00122-020-03579-z (PMC7311499; doi:10.1007/s00122-020-03579-z)
Supplement: Supplementary file 3 — Supplementary file3 (DOCX 41 kb) [file 122_2020_3579_MOESM3_ESM.docx]

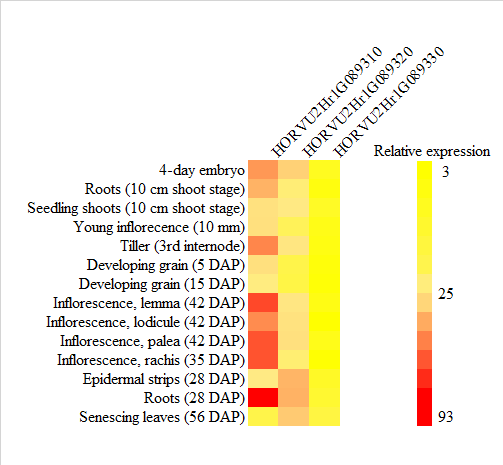


**Fig. S3** Relative gene expression in FPKM for each putative gene located in *qGL2H* averaged across three biological replicates for 14 different developmental tissues of barley cv. Morex. DAP; days after pollination
